# The Tellurium compound, AS101, increases SIRT1 level and activity and prevents type 2 diabetes

**DOI:** 10.18632/aging.100468

**Published:** 2012-06-30

**Authors:** Meital Halperin-Sheinfeld, Asaf Gertler, Eitan Okun, Benjamin Sredni, Haim Y. Cohen

**Affiliations:** The Mina and Everard Goodman Faculty of Life Sciences, Bar-Ilan University, Ramat-Gan 52900, Israel

**Keywords:** AS101, SIRT1, T2D

## Abstract

The histone deacetylase, SIRT1, plays a major role in glucose regulation and lipid metabolism. Ammonium Trichloro (dioxoethylene-o,o') Tellurate, AS101, is a potent in vitro and in vivo immunomodulator, with several potential therapeutic applications. AS101 administration resulted in upregulation of SIRT1 protein expression and activity. These effects were associated with decreased levels of serum insulin like growth factor-1 (IGF-1) and of insulin. The properties of AS101 prompted us to investigate its potential therapeutic role in rats with type 2 diabetes (T2D). T2D was induced by a high fat diet combined with a low dose of Streptozotocin (STZ). Treatment with AS101 before manifestation of hyperglycemia, resulted in increased insulin sensitivity, and decreased blood glucose levels, and prevented symptoms of diabetes including defective glucose clearance, fatty liver, and abnormal distribution of insulin-producing beta cells in the pancreas. Treatment after disease emergence resulted in partial restoration of normal glucose homeostasis. Diabetic rats showed a reduction in liver SIRT1 levels. In both treatment regimens the reduction in SIRT1 levels in the liver were blocked by AS101 consumption. Together, these findings demonstrate the therapeutic potential of AS101 for treating T2D, and for reversing impaired fat and glucose metabolism.

## INTRODUCTION

The incidence of Type 2 diabetes (T2D) depends on complex interaction of genetic and environmental factors [1-3]. T2D is characterized by chronic irregular lipid and carbohydrate metabolism followed by insulin resistance in target tissues [1] and increasing to epidemic proportions [4]. Insulin lowers blood glucose levels by facilitating glucose uptake, mainly into skeletal muscle and fat tissue, and by inhibiting endogenous glucose production in the liver. However, insulin resistance occurs when a normal dose of insulin is incapable of eliciting these metabolic responses [5]. In this progressive disease, susceptible individuals develop impaired peripheral tissue responses to insulin and compensatory hyperinsulinemia. In many patients, β-cells fail to secrete sufficient amounts of insulin to compensate for insulin resistance, and they therefore fail to maintain normal glucose levels, marking the onset of diabetes. In a significant portion of Type 2 diabetic patients, the disease progresses to a complete loss of β-cell insulin secretion [6], thus necessitating exogenous administration of insulin, in addition to other medications, to achieve adequate glycemic control [7, 8]. It has been proposed that persistent insulin resistance accelerates β-cell failure. Therefore, earlier intervention to correct insulin resistance and/or to protect β-cells may prevent negative development and progression of the disease [9, 10].

A recent series of studies in several organisms revealed multiple important functions of the sirtuin family of proteins in energy metabolism [11]. Sirtuins are highly conserved NAD+ dependent deacetylases [12, 13]. The mammalian sirtuins, SIRT1-SIRT7, are implicated in a number of cellular and physiological functions including gene silencing, stress resistance, apoptosis, mitochondrial function, energy homeostasis, and extension of lifespan. Anti-diabetic drugs inhibit obesity-linked phosphorylation of PPARgamma by Cdk5 [14, 15]. SIRT1 also promotes cell survival and inhibits apoptotic cell death by deacetylating the p53 [16], Ku70 [17], and fork-head transcription factors [18-20]. Therefore, SIRT1 is considered a key regulator of cell survival under various stressful conditions [21, 22]. Among its multiple reported targets, SIRT1 deacetylates and thereby activates PGC1α [23], an essential cofactor in mitochondrial biogenesis, regulating metabolic rate. Other studies have shown that SIRT1 represses Peroxisome Proliferator-Activated Receptor γ (PPARγ) function, increasing lipolysis in white adipose tissue [24]. SIRT1 was also shown to act as a regulator of insulin secretion in pancreatic β-cells by repressing uncoupling protein 2 [25] and its levels were inversely correlated with insulin and insulin like growth factor IGF-1 [26]. Another novel function of SIRT1 overexpression is the protection of pancreatic β-cells from cytokine toxicity [27].

An organotellurium compound previously developed in our laboratory, Ammonium Trichloro (dioxoethylene-o,o') Tellurate, AS101, is a potent *in vitro* and *in vivo* immunomodulator [28-30]. This non-toxic compound has been shown to have beneficial effects in diverse preclinical and clinical studies. Accumulated research suggests that much of the biological activity of organotellurium compounds is directly related to their specific chemical interactions with endogenous thiols [31]. Such tellurium thiol compounds may be important for the manifestation of the biological function or for transportation of the tellurium species to its target location. In a previous study, we clarified several mechanistic aspects of this chemistry and discussed its relationship to the biological activity of AS101 [32]. AS101 exhibits diverse biological effects; some of its activities have been primarily attributed to the direct inhibition of the anti-inflammatory cytokine, IL-10 [33]. Other features of AS101, including regulation of growth factors levels [34] and immunomodulatory activity in different systems [35], give the compound its therapeutic activity.

In this study, the role of AS101 on SIRT1 activity and T2D progression was examined. T2D was generated by the STZ+HFD rat model which imitates the disease development in humans. The HFD disrupts metabolism and causes insulin resistance, which results from a chronic disruption in carbohydrate and lipid metabolism, and plays a major role in the progression and pathogenesis of T2D. Therefore, HFD sensitizes pancreatic β cells to low doses of STZ [36].

Here, we characterized the ability of AS101 to enhance the expression and activity of the SIRT1 protein. Those effects may protect cells from physiological injury caused by the metabolic syndrome in a rat model of T2D induced by high fat diet (HFD) and low dose of streptozotocin (STZ).

Thus, activation of SIRT1 by AS101 may signify a promising strategy for prevention and treatment of metabolic syndrome.

## RESULTS

### AS101 affects SIRT1 expression and activity

**Figure 1:**
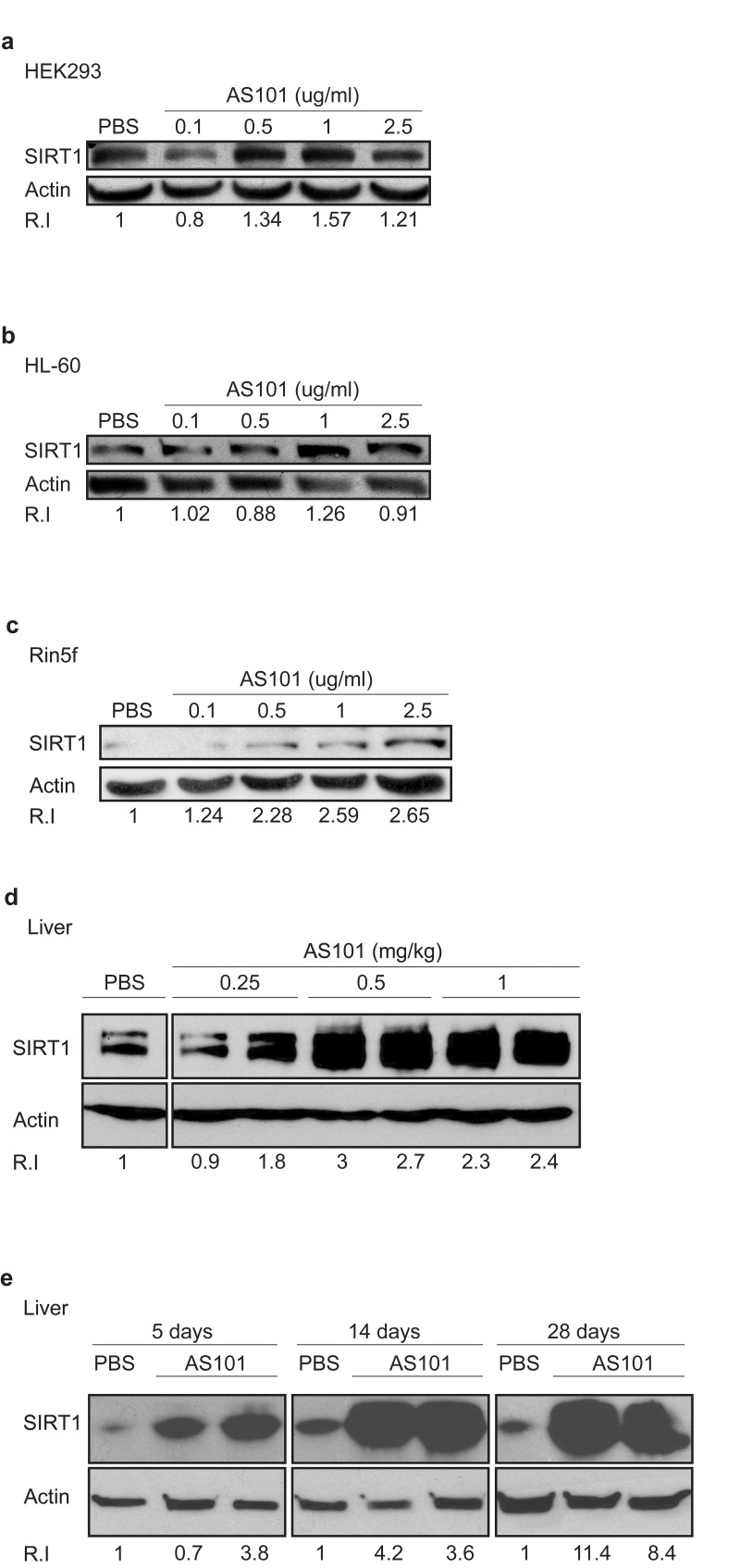
AS101 increases SIRT1 levels *in vitro* and *in vivo* (**A-C**) AS101 increases SIRT1 expression in several cell lines. For in vitro studies, AS101 and PBS were added to cultures of HEK293 (**A**), HL-60 (**B**) and Rin-5f (**C**) cells at a concentration of 0.1-2.5μg/ml, for 48 hours. Each experiment was done in three independent replicates and representative blots are shown. (**D-E**) AS101 increases SIRT1 expression in healthy rat livers. For the in vivo assay, healthy rats were injected daily i.p with AS101 or PBS at different concentrations for 14 days (0.25-1mg/kg AS101(**D**)) and for different periods of time (0.5 mg/kg) (**E**) (n=4 for each group.) SIRT1 levels in protein extracts from cell lines or rat liver, were measured by western blot analysis with anti- SIRT1 antibodies; actin was used as a loading control.

The effect of the telluric compound AS101 on SIRT1 protein expression was first examined in an *in vitro* assay using cell lines. AS101 induced SIRT1 expression in a dose dependent manner in three different cell lines, HEK293, HL-60 and Rin-5f (Figure 1a-c). Similar to other therapeutic compounds, AS101 also exhibits an optimal range, beyond which the effective activity is reduced. The effect of AS101 on SIRT1 protein levels was next examined in healthy rats treated with AS101 or PBS for 14 days. AS101 treated rats showed a large increase in SIRT1 protein levels in the liver (Figure 1d) and kidney extracts of rats treated with AS101 (Supplementary 1a). In order to determine the minimal AS101 treatment time that results in SIRT1 induction, SIRT1 levels were measured at different times after AS101 injection. Significant induction of SIRT1 expression was already seen after 5 days of AS101 treatment (Figure 1e). These results show that AS101 treatment significantly induces SIRT1 protein expression *in vitro* and in *vivo*.

**Figure 2:**
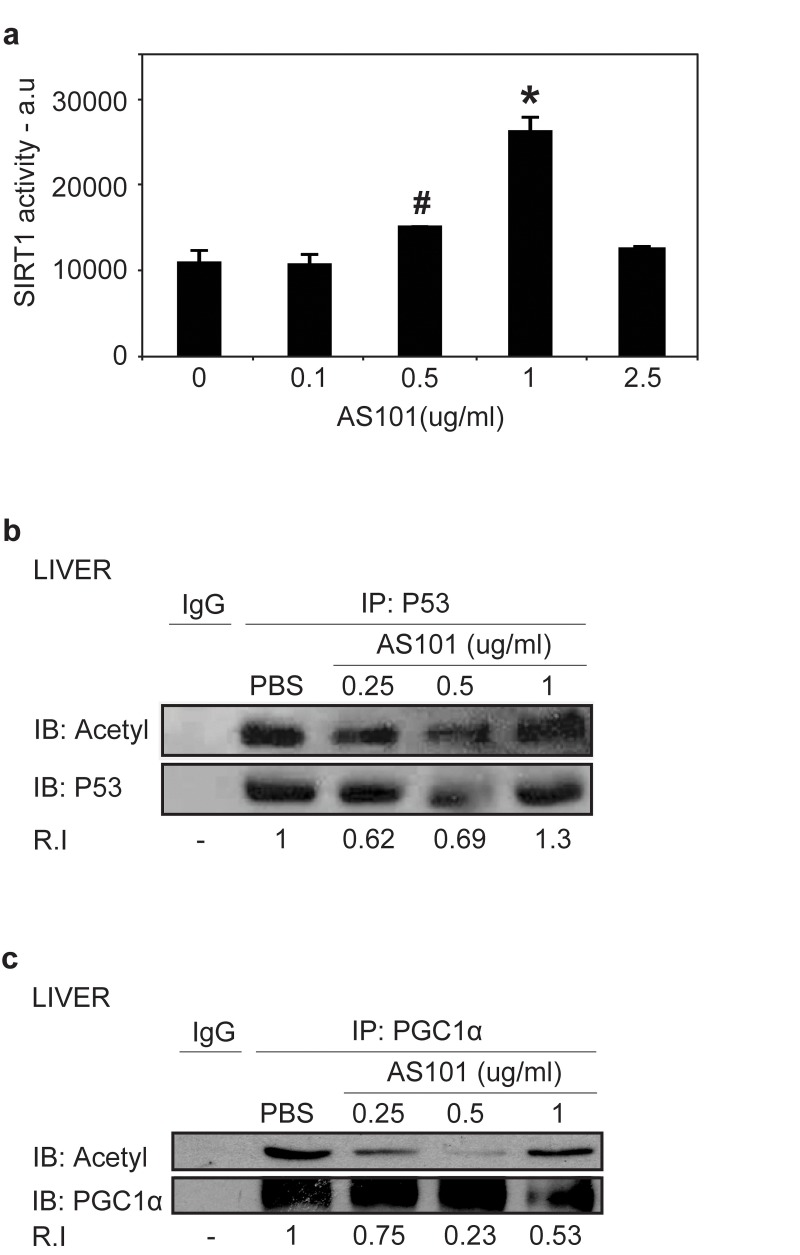
AS101 increases SIRT1 protein activity *in vitro* and *in vivo* (**A**) AS101 induces activity of recombinant SIRT1. To determine in vitro SIRT1 activity, recombinant SIRT1 was incubated with AS101 at different concentration (0.1-2.5 μg/ml) or with PBS for 1 hour. Activity was examined based on SIRT1 de-acetylation of a fluorogenic acetylated peptide substrate. Results shown are average ±SEM of three independent experiments (#p=0.05,*p<0.05 versus PBS). (**B**-**C**) AS101 treatment reduces the acetylation levels of SIRT1 substrates. In vivo SIRT1 activity was measured by de-acetylation of its substrates PGC1α (**B**), and p53 (**C**) in rat liver extracts from treated for 14 days with AS101 or PBS at different concentrations. To detect acetylation levels, immunoprecipitated PGC1α and p53 were immunoblotted with antibodies against themselves and against pan acetyl antibodies. IgG was used as a control for the immunoprecipitation. Results shown are representative of the experiments from three independent replicates that gave similar results.

To determine if the increase in SIRT1 levels upon AS101 treatment is reflected in an increase in SIRT1 enzymatic activity, several approaches were taken. First, the direct activity of AS101 on recombinant SIRT1 was examined *in vitro*. SIRT1 activity was measured by a deacetylation assay using fluorogenic acetylated peptide, representing human p53, which is a known SIRT1 substrate [37]. A significant increase in SIRT1 activity was observed (p<0.05) (Figure 2a). Next, the acetylation levels of two known SIRT1 substrates, p53 and PGC1α [38], were tested *in vivo*, in tissue samples from rats treated with AS101 or PBS. Tissue extracts from rats treated with 0.25 and 0.5 mg/kg AS101 for 14 days, exhibited significantly reduced levels of acetylation relative to extract from PBS treated control animals (Figure 2b-c). This decrease may partially reflect the higher expression of SIRT1 protein in AS101 treated animals. Therefore, together with the *in vitro* examination, we suggest that SIRT1 activity was increased and AS101 treatment is capable of enhancing SIRT1 expression and deacetylation activity.

### The mechanism for the increase in SIRT1 expression in response to AS101

**Figure 3:**
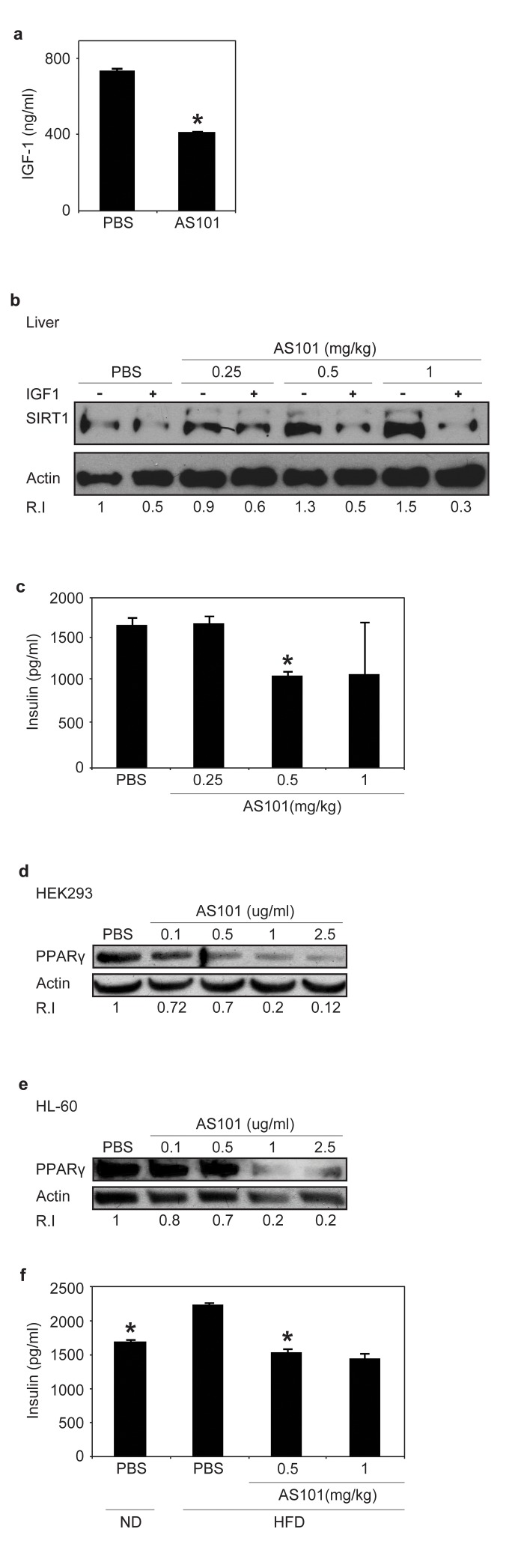
AS101 treatment increases SIRT1 expression through a possible mechanism of serum IGF-1 reduction (**A**) Sera from AS101 treated rats contained significantly reduced IGF-1 levels. AS101 (0.5mg/kg) or PBS was injected daily i.p into healthy rats for 14 days and IGF-1 levels were measured by ELISA (*p<0.01 versus PBS). Error bars represent the ±SEM of all animals (AS101 n=5, PBS n-4). (**B**) SIRT1 expression increased in HEK293 cells incubated with AS101-treated rat serum. Supplementing with rIGF-1 back to normal levels (500 ng/ml) restored SIRT1 levels as in control mice. Cell extracts, were used for western blot analysis with anti- SIRT1 antibodies; Actin was used as a loading control. (**C**) Sera from AS101 treated rats contained significantly reduced insulin levels. AS101 (0.25-1mg/kg) and PBS were injected daily i.p to healthy rats for 14 days. Serum insulin levels were measured by ELISA kit after 4 hours starvation (*p<0.001 versus PBS) (n=3 in each group). (**D**-**E**) AS101 decreases PPARγ levels in tissue culture in vitro. AS101 and PBS were added to tissue cultures of HEK293 (**D**) and HL-60 (**E**) cells at the indicated concentrations for 48 hours. Western blots were probed with PPARγ antibody; actin was used as a loading control. (**F**) AS101 maintains normal insulin levels in HFD fed rats. AS101 (0.5-1mg/kg) and PBS was injected daily i.p into HFD/ND rats for 14 days. Serum insulin was measured by ELISA after 4 hours of fasting (*p<0.001 versus HFD+PBS, n=3 in each group). Error bars represent the SEM. Results shown in **B**, **D** and **E** are representative of three independent replicates that gave similar results (using different batches of rat sera).

Previous studies indicated an inverse relationship between serum levels of insulin-like growth factor 1 (IGF-1) and SIRT1 expression in rats fed a calorie restricted (CR) diet [26], in an unknown mechanism. To explore the mechanism underlying the increase in SIRT1 expression, the effect of AS101 on IGF-1 levels were tested. Treatment with AS101 resulted in a significant reduction of serum IGF-1, compared to PBS control treatment (p<0.001) (Figure 3a). These findings show that AS101 treatment mimics the influence of CR on IGF-1 and SIRT1 levels.

To investigate whether IGF-1 or another mediator is responsible for the effect of AS101 on SIRT1 protein expression, a cell culture model was used. Human embryonic kidney (HEK) 293T cells were cultured in the presence of serum from AS101 or PBS treated rats. SIRT1 expression was significantly higher in HEK293 cells grown in the presence of AS101 treated rat serum compared to cells grown in serum from PBS treated rats (Figure 3b). Supplementing cells grown in AS101 sera with recombinant IGF-1 to a concentration equal to the IGF-1 concentration in the normal rat sera resulted in reduced SIRT1 protein expression (Figure 3b). These results suggest that AS101 increases SIRT1 protein expression, at least in part, by reducing IGF-1 levels.

### The effect of AS101 on SIRT1 metabolic pathways

Similar to IGF-1 levels, previous studies have described an inverse correlation between serum levels of SIRT1 and insulin, which is a possible mechanisms through which SIRT1 regulates metabolic pathways [26]. Insulin levels were therefore examined in the rat sera after daily treatment with AS101 (0.25-1mg/kg) or PBS for 14 days. At a dose of 0.5 mg/kg AS101, treated rats showed 35% lower insulin levels versus the PBS treated rat sera (p<0.001) (Figure 3c). Picard et al., have shown that SIRT1 activation, promotes lipolysis and a reduction in fat accretion, due to repression of PPARγ. [24]. In order to examine if AS101 affects additional SIRT1 dependent metabolic pathways, the level of Peroxisome Proliferator Activated Receptor Gamma (PPARγ) was tested. Incubation of HEK293 and HL-60 cell lines with AS101 (0.1-2.5μg/ml) resulted in a dose dependent reduction in PPARγ protein expression relative to the control cells (Figure 3d-e). Incubation with AS101 reduced PPARγ expression in parallel to increased SIRT1 expression. The effects of AS101 on several important metabolic factors, led us to investigate the effects of AS101 on insulin resistance.

### AS101 prevents development of insulin resistance *in vivo*

Treatment with AS101 reduced insulin levels and induced SIRT1 expression and activity, leading us to examine whether AS101 treatment can also mimic the reported inhibition of increased insulin levels in SIRT1 overexpressing or activated rodents fed with a high fat diet (HFD) [39, 40].

Normal rats were fed with HFD with AS101 or PBS for 14 days. In comparison to control rats fed with HFD and treated with PBS, serum insulin levels were significantly lower by 30% and 35% in rats treated with AS101 at concentrations of 0.5 and 1 mg/kg respectively (Figure 3f). Moreover, AS101 treatment also prevented development of insulin resistance (data not shown). Thus, AS101 affects SIRT1 related metabolic pathways by changing the insulin levels.

### AS101 treatment prevents T2D in the HFD+STZ rat model

**Figure 4:**
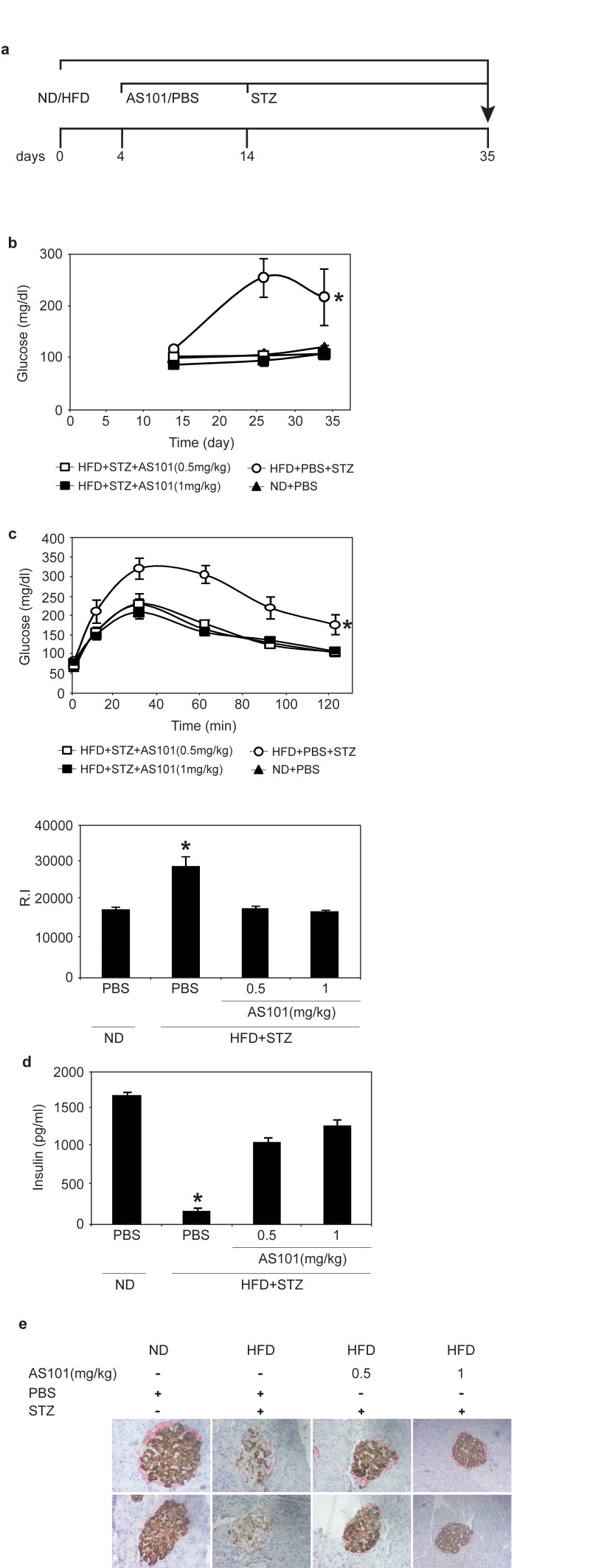
AS101 reverses HFD+STZ induced T2D (**A**) Course of experiment. (**B**) AS101 treatment prevents glucose increase in HFD+STZ induced T2D rat model. (*p<0.01 all groups vs. HFD+PBS group, n=6 in each group). (**C**) AS101 treatment maintains glucose tolerance. On day 17 (following the onset of diabetes) rats were fasted for 12 hours before i.p injection of 2mg/kg glucose. (*p<0.001 versus HFD+PBS+STZ, n=6 in each group). The histogram represents the incremental area under the glucose curve. (**D**) AS101 treatment maintains proper insulin levels in the serum of rats after 4 hours starvation (*p<0.001 all groups versus HFD+PBS+STZ, n=6 for each group). (**E**) AS101 preserves insulin production in the pancreas. Immunohistological staining for insulin (brown) and glucagon (red) of the rat pancreas. Pictures represent two out of the six animals in each group. Error bars represent the ±SEM.

Next, we tested the ability of AS101 to prevent T2D in a rat model. Diabetes was induced by a combination of HFD and a single low dose Streptozotocin (STZ) injection, which is toxic to insulin-producing beta cells [36]. The rats were fed a high fat diet (HFD) for the whole experiment period, and two weeks into the experiment the rats were injected with an intraperitoneal injection of a low dose of STZ (35 mg/kg) (see figure 4a for experimental design). The effect of AS101 was first examined when administered before the onset of hyperglycemia. This experiment included four groups: Control group of normal diet (ND) fed rats injected with PBS (ND + PBS) (group 1), Diabetic groups of HFD fed rats with single dose STZ divided to treatment injected with PBS as control (HFD + STZ + PBS) (group 2), AS101 treatment in two concentrations 0.5 mg/kg (group 3) or 1 mg/kg (group 4) (HFD + STZ + 0.5 mg/kg AS101 and HFD + STZ + 1 mg/kg AS101 respectively). AS101 treatment started four days after the beginning of the HFD and was injected daily for 14 days and then continued every other day. As seen in Figure 4b, pretreatment with AS101 prevented the increase in blood glucose (P<0.01), and preserved optimal glucose tolerance (Figure 4c) and insulin levels (p<0.001) (Figure 4d). In addition, immunostaining of insulin and glucagon indicated that treatment with AS101 preserved normal insulin production by the pancreatic β cells and maintained the islets structure [41] (Figure 4e). Finally, AS101 treatment reduced serum triglycerides to a normal level (data not shown). Thus, AS101 treatment prevents hyperglycemia associated with T2D and some of the symptoms of the disease in the HFD+STZ rat model.

**Figure 5:**
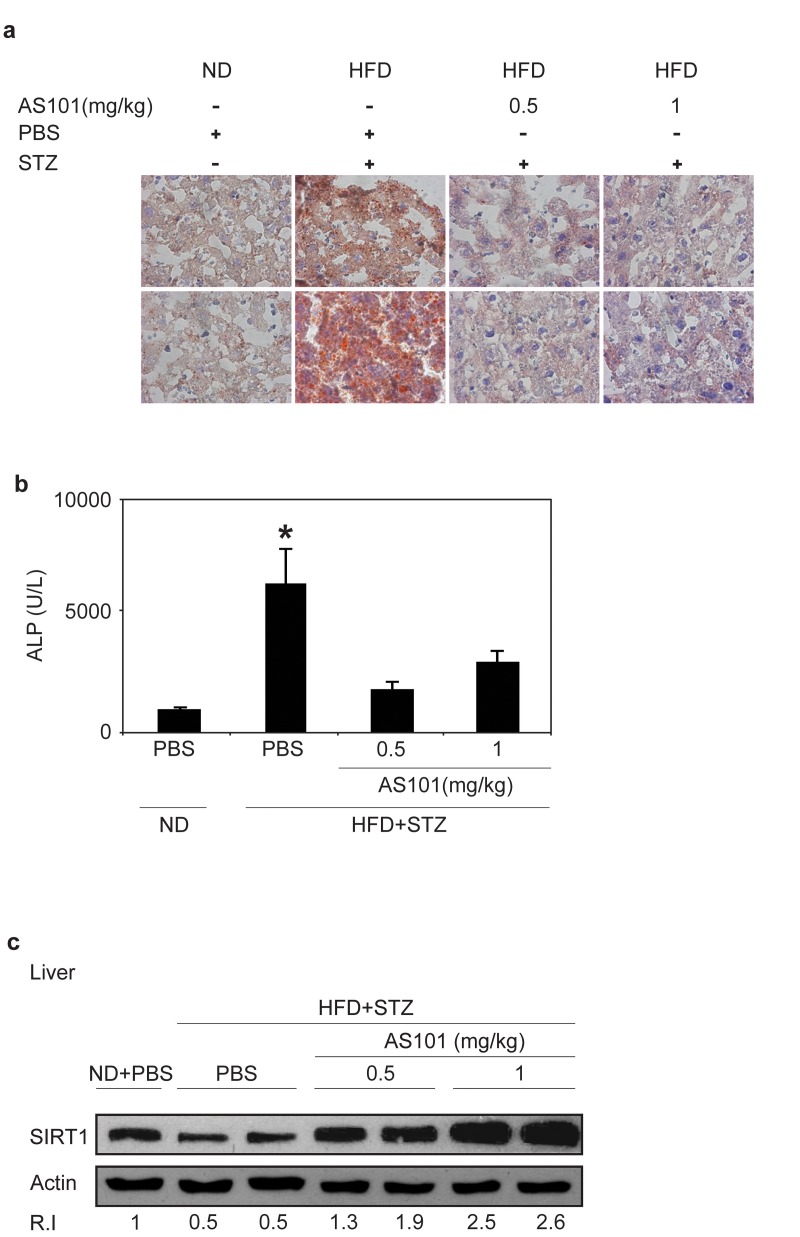
AS101 treatment protects rats from HFD+STZ induced hepatosteatosis (**A**) Oil red staining of lipid droplets in frozen liver sections. Pictures represent two out of the six animals in each group. (**B**) AS101 treatment reduces serum ALP levels. (*p<0.05 decrease vs. diabetic group, n=6 in each group).

Chronic HFD causes an accumulation of lipids in the liver leading to fatty liver disease [42] and SIRT1 was recently shown as a potential therapeutic target for treatment fatty liver disease [43, 44]. Therefore, frozen liver sections were stained with Oil Red to follow the effect of AS101 on hepatic accumulation of lipids. AS101 (0.5 and 1 mg/kg) treatment protected rats from HFD+STZ induced hepatosteatosis and retained healthy tissue (Figure 5a). In agreement with these findings, Alkaline Phosphatase (ALP), a marker of liver damage, was examined. ALP levels increased seven fold in the serum of HFD + STZ diabetic rats; these increases were blocked in AS101 treated rats and ALP levels remained similar to the ALP levels of normal rats (p<0.05) (Figure 5b). Further, in agreement with Peng et al, [45] the baseline levels of SIRT1 were decreased in the liver of mice treated with HFD and STZ (Figure 5c), in contrast, treatment with AS101 restored to normal or elevated the levels of SIRT1 protein in comparison to the diabetic rats (Figure 5c). These findings show that AS101 treatment started before the manifestation of hyperglycemia in the HFD+STZ T2D model prevented onset of symptoms.

### Treatment of T2D with AS101 in the HFD+STZ rat model

**Figure 6:**
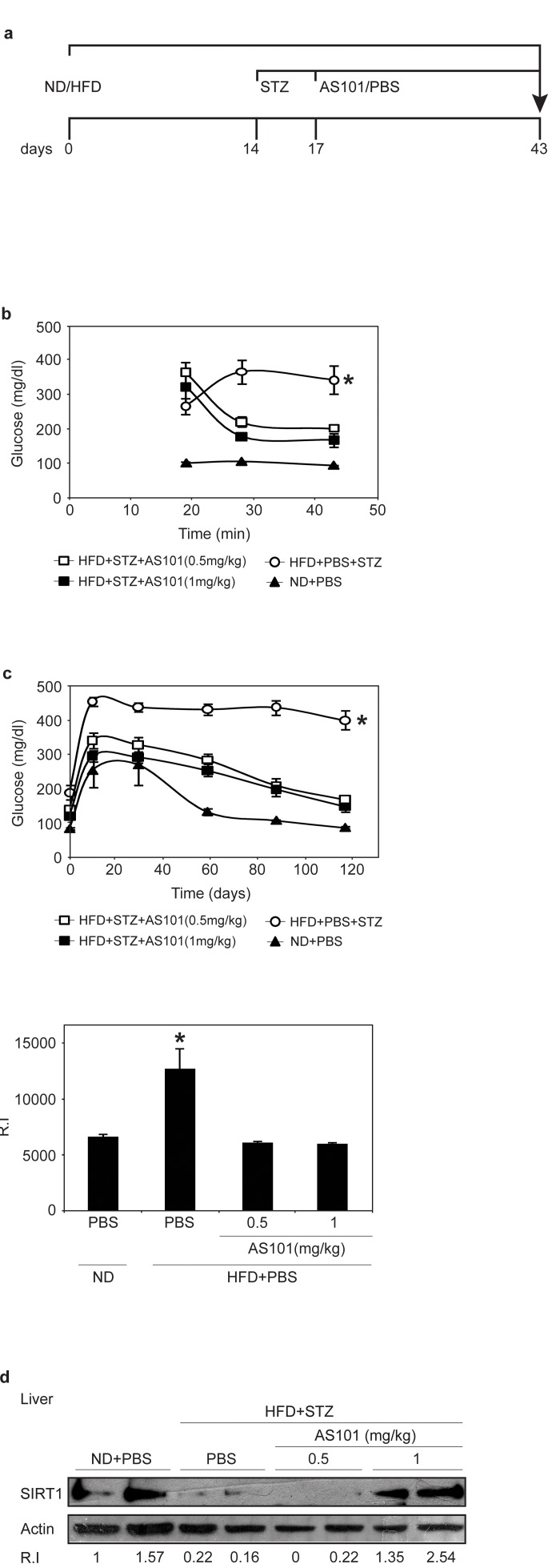
AS101 treatment after disease induction results in partial beneficial effects in the HFD+STZ T2D rat model (**A**) Course of experiment. (**B**) AS101 reduced glucose levels in HFD+STZ T2D rat model. Glucose levels were measured in peripheral blood at different times (*p<0.05 all groups vs. HFD+PBS group, n=6 in each HFD group, n=3 in ND group). (**C**) AS101 treatment restores proper glucose tolerance. Rats fasted for 12 hours before i.p injection of 2mg/kg glucose. (*p<0.01 versus HFD+PBS+STZ, n=6 in each group). The histogram represents the incremental area under the glucose curve. (**D**) AS101 (1 mg/kg) treated rats show higher SIRT1 protein levels in liver extract prepared at the end of the experiment. Western blot analysis was done with anti- SIRT1 antibodies; actin was used as a loading control. Error bars represent the ±SEM.

The effect of treatment with AS101 after the onset of disease (glucose >250 mg/dL) was also tested. Here, AS101/PBS treatments were given after 17 days of HFD and 3 days after a single low dose of STZ (Figure 6a). AS101 treatment of diabetic rats resulted in significant beneficial effects. As shown in Figure 6b, after only 3 days of AS101 treatment, blood glucose levels were significantly reduced (p<0.05). Glucose tolerance test (GTT) assay, demonstrated that AS101 significantly (p<0.01) improved the glucose clearance in HFD+STZ treated rats to a level similar to that of the PBS treated rats under regular diet (Figure 6c).

Finally, these beneficial effects were accompanied by increases in SIRT1 protein expression in rats treated with 1 mg/kg AS101 (Figure 6d).

Taken together, these results suggest the use of AS101 as a novel pharmacological means to regulate metabolic disorders by increasing SIRT1 levels and activity, and interfering with T2D development and progression.

## DISCUSSION

This study demonstrates that AS101 can decrease the pathological symptoms and block the development of T2D disease in rat model. We also showed that AS101 enhances SIRT1 expression and activity. Furthermore, AS101 exhibited the most significant improvements of T2D when treatment was given at the onset of diabetes induction, whereas treatment after the onset of the disease resulted in partial restoration of normal glucose metabolism. We further demonstrated an association between the ability of AS101 to increase SIRT1 protein levels and the beneficial effects on glucose metabolism [46-49].

In some animal models of T2D, SIRT1 protein levels decrease [50-52], suggesting a role for SIRT1 in regulating T2D. In support to this notion, mice overexpressing SIRT1 are protected from the metabolic damage of HFD, including the development of T2D, via induction of antioxidants and stimulation of PGC1alpha [39]. Thus, this unique affect of AS101 on SIRT1 protein *in vivo* and *in vitro* may lead to a new therapeutic approach [49]. The gradual progression of T2D in patients [53] suggests the possibility of therapeutic intervention at several stages of the disease process. For example, by regulating metabolic disorders via increasing SIRT1 levels to inhibit disease progression. The results described in this study, signify the clinical value of AS101 in treating T2D. Moreover, whereas most SIRT1 activating agents described to-date affect only protein activity, this study shows that AS101, a non toxic compound, increases both SIRT1 protein expression and activity. Thus, the therapeutic potential of AS101 against T2D is at least similar to those achieved by other SIRT1 activators.

We suggest that SIRT1 is responsible to the protective effect of AS101 in the current rat model of T2D. Similar findings were observed in transgenic mice over-expressing SIRT1 that were protected against obesity dependent impaired glucose tolerance [39]. Importantly, other features of AS101 may also mediate the beneficial activities of the compound. AS101 may influence the metabolic syndrome through interference in insulin pathways. In addition, anti inflammatory and anti apoptotic properties [32, 35, 54] of the compound may potentially enable it to preserve β cells.

The insulin signal cascades, which include PKB/Akt through PI3K and mTOR, stimulate S6K1, resulting in a negative feedback, which may cause insulin resistance [55, 56]. AS101 was shown to inhibit PKB/Akt in a model of multiple myeloma [57]. This inhibition may be mediated through reduction in the levels of growth factors [34], analogous to the reduction of insulin or IGF-1, as was shown here.

In addition, AS101 has an anti apoptotic effect due to its selective inhibition of cysteine protease proteins, notably caspases 1, 3, and 9 *in vitro* and *in vivo* [31-33, 54, 58]. Caspase1 inhibition provides the compound with anti inflammatory properties by repressing specific inflammation cytokines including IL1-β, IL-18 and TNFα. [59]. IL1-β and TNFα induce IRS-2 phosphorylation, which leads to insulin resistance, followed by the apoptotic death of β cells [60, 61]; the inhibition of cytokines by AS101 may contribute to β cell preservation. In this study, AS101 treatment indeed preserved β cells as shown by immunohistochemical staining (Figure 4e). In addition to the inhibition of inflammatory cytokines, AS101 can positively affect β cell production via its known immunomodulator function, which reduces IL-10, and thereby enhances GDNF production [32, 35, 62]. GDNF increases β cell production and prevents their apoptosis in an STZ model [63].

Numerous studies have been performed to identify a compound that promotes SIRT1 protein activity only [64-66]. In comparison to previously published SIRT1 activators, AS101 have several unique properties. First, AS101 enhance the expression and activity of the SIRT1 whereas the other activators enhanced SIRT1 activity only. Second, no side effects were found for AS101 while other SIRT1 activators results in significant side effects when supplemented in high concentrations [67-69].

Here, we suggest that AS101, a non toxic compound currently in clinical trials for other indications [70, 71], may be effective as a treatment for T2D by enhancing SIRT1 protein expression and activity.

Understanding the mechanisms and actions of AS101 in T2D, including the elucidation of the role of SIRT1 upregulation as well as additional properties of AS101 that might evoke beneficial effects, may contribute to the development of new pharmacological means to control this devastating disease.

## MATERIALS AND METHODS

### Cell cultures and experimental treatment

HEK293 cells were maintained in DMEM (Biological Industries, Beit Haemek, Israel) containing 10% FBS (Biological Industries, Beit Haemek Israel) and 1% Pen/strep (100U/ml) (Biological Industries, Beit Haemek, Israel).

HL-60 and Rin5f were maintained in RPMI (Biological Industries, Beit Haemek Israel) containing 10% FBS and 1% Pen/strep (100U/ml) at 370C in a 5% CO2 humidified atmosphere. In some of the experiments, FBS was replaced with serum from AS101/PBS treated rats; in those experiments the cells were plated with commercial serum, and serum replaced after the cells adhered. After serum replacement, cells were incubated for 48 hours, and were then lysed with a lysis buffer (1M Tris (pH=7.4), 1.5M NaCl, 1% Triton-X, 10% Glycerol, 50mM EDTA (pH=8), 0.1M Sodium vanadate, 0.1M PMSF, 0.1% protease inhibitor cocktail (Calbiochem, San Diego, CA, USA)).

### PGC1α and p53 acetylation assays

PGC1α and p53 lysine acetylation was analyzed by immunoprecipitation followed by western blot using an antibody specific for acetyl lysine (Cell signaling, Danvers, MA, USA). Liver extracts of AS101 or PBS treated rats after 14 days of treatment were immunoprecipitated using anti-PGC1α and anti-p53 antibodies. The immunoprecipitated complex was divided into equal aliquots and further immunoblotted with antibodies against the acetylated residue, stripped and reblotted against the substrate. IgG of irrelevant specificity was used as a control for the immunoprecipitation.

### Immunoblotting

Samples were boiled for 5 minutes, electrophoresed on 8% SDS-PAGE, transferred to nitrocellulose, and immunoblotted with specific Abs (SIRT1, p53, PGC1-α, and PPARγ, Santa Cruz (Heidelberg, Germany); actin, HRP (Sigma Aldrich (St. Louis, MO, USA)). Blots were developed using horseradish peroxidase-conjugated secondary Ab's and the ECL detection system (Thermo scientific Pierce protein research protein products, Rockford, IL, USA).

Animal Experiments. Male Sprague Dawley rats (160-180gr) were purchased from Harlan Laboratories (Jerusalem, Israel). Animal experiments were performed in accordance with approved institutional protocols and all experiments were approved by the Institutional Animal Care and Use Committee.

### T2D model in rats

The rats were allocated into two dietary regimens by feeding them either a normal diet (ND, n=6 animals) or high fat diet (HFD, n=18) (60% fat) ad libitum, respectively, for a period of 1 month. After 2 weeks of dietary manipulation, the HFD fed rats were injected intraperitoneally (i.p) with a low dose of STZ, Calbiochem (San Diego, CA, USA) (35 mg/kg). The rats were maintained on their respective diets until the end of the study. AS101 (0.5 or 1 mg/kg) or PBS were injected i.p daily for 14 days, and then every 2 days. The T2D model last for 34 days, and treatment with AS101 or PBS started from the fourth day of the high fat diet (Figure 4a) or for 43 days with AS101 or PBS starting on day 17 (Figure 6a).

### Blood glucose level, GTT

After 12 hr fast, rats received an i.p injection of 2 g/kg glucose, Sigma Aldrich (St. Louis, MO, USA). Blood glucose concentrations were measured via tail bleed at the indicated times before and after the injections. All glucose measurements were performed using a glucometer, Free Style Freedom (Alameda, CA, USA).

### Protein level/activity

SIRT1 activity levels were determined using recombinant SIRT1 (b; SIRT1 was incubated with AS101 or PBS at different concentrations for 1 hour. Activity was measured using the SIRT1 Fluorometric Activity Assay/Drug Discovery Kit Biomol (Exter, United Kingdom). IGF-1 levels were determined in the serum of AS101/PBS treated rats using Active IGF-1 Kit, DSL (Webster, Texas, USA). Insulin levels were determined in treated rat serum using a rat/mouse insulin ELISA kit, Millipore (Billerica, MA, USA).

### Metabolic measurements

ALP was determined at the Veterinary Institute service in Beit Dagan, Israel.

### Oil Red O staining

Frozen liver was sectioned serially at 4μm thickness with a cytostat, placed on slides, and dried for 15 min at 37°C. Sections were then fixed for 10 min in neutral buffered 10% formalin. To detect neutral lipid accumulation, sections were stained with Red Oil 'O' for 10 min, counterstained with hematoxylin for 2 min, and coverslipped with a water-based mounting medium.

### Insulin and glucagon staining

Pancreatic paraffin sections were prepared and stained with Picture – double staining kit- insulin and glucagons, Zymed Laboratories, Inc. (San Francisco, CA, USA).

### Statistical Analysis

For repeated experiments, data are presented as means +/−S.E.M. Statistical analysis was performed using a two tailed unpaired Student's t-test. P<0.05 or 0.01 (as specified) was considered to be statistically significant. For several time course experiments, ANOVA statistical analysis was performed.

## SUPPLEMENTARY FIGURE

Supplementary Figure 1 AS101 increases SIRT1 levels in rats kidneys AS101 increases SIRT1 expression in healthy rat kidneys. For *in vivo* assay, healthy rats were injected daily i.p with AS101 (0.5 mg/kg) or PBS for 14 days. (n=4 for each group) kidney extracts, were used for western blot analysis with anti- SIRT1 antibodies; actin was used as a loading control.

## Abbreviations

T2D: Type 2 diabetes
IGF-1: Insulin like growth factor 1
STZ: Streptozotocin
PPAR γ: Peroxisome Proliferator Activated Receptor γ
