# Supplementary figures and images for: The Tellurium compound, AS101, increases SIRT1 level and activity and prevents type 2 diabetes
Source: Aging (Albany NY). 2012 Jun 30;4(6):436–47. doi: 10.18632/aging.100468 (PMC3409680; doi:10.18632/aging.100468)

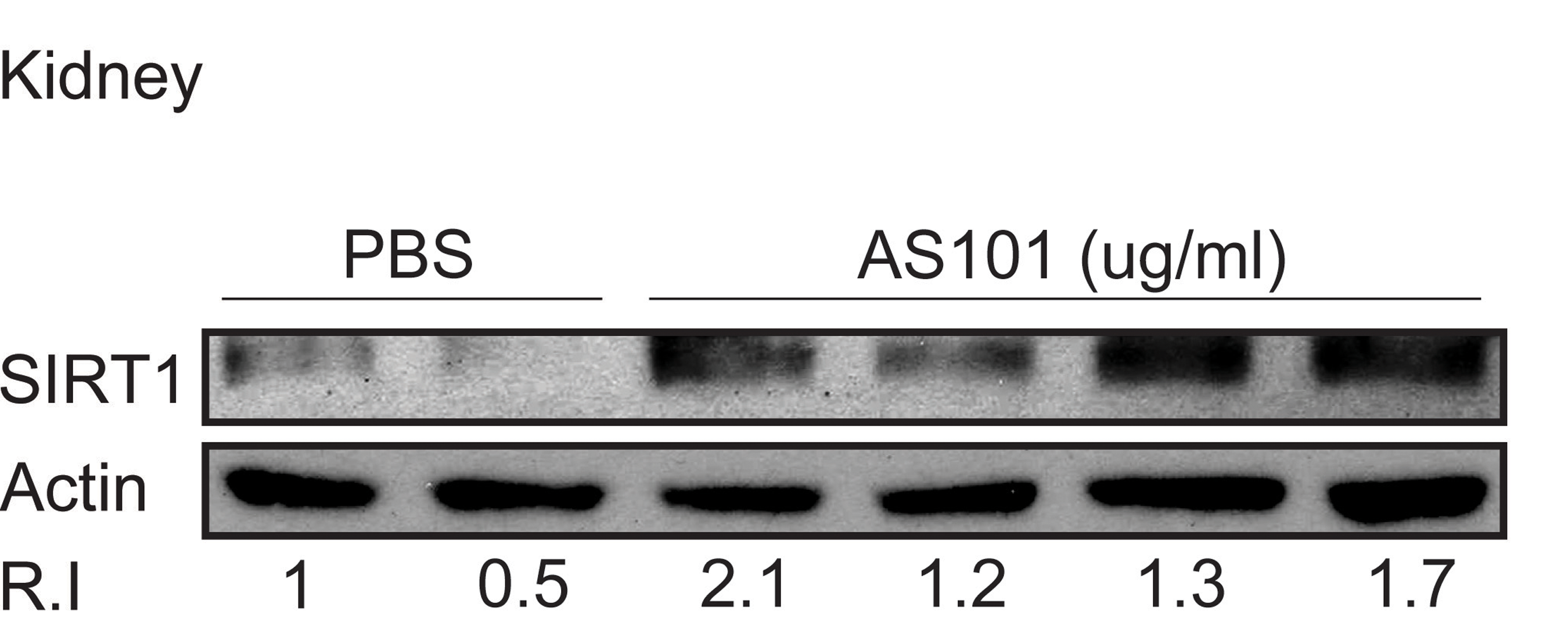

Supplement: Supplementary Figure 1 — AS101 increases SIRT1 expression in healthy rat kidneys. For in vivo assay, healthy rats were injected daily i.p with AS101 (0.5 mg/kg) or PBS for 14 days. (n=4 for each group) kidney extracts, were used for western blot analysis with anti- SIRT1 antibodies; actin was used as a loading control. [file aging-04-436-s001.tif]
